# Effects of Substrate Preheating Temperatures on the Microstructure, Properties, and Residual Stress of 12CrNi2 Prepared by Laser Cladding Deposition Technique

**DOI:** 10.3390/ma11122401

**Published:** 2018-11-28

**Authors:** Chenggang Ding, Xu Cui, Jianqiang Jiao, Ping Zhu

**Affiliations:** School of Materials Science and Engineering, Dalian Jiaotong University, Dalian 116028, China; jjq053190@163.com (J.J.); zhuping@djtu.edu.cn (P.Z.)

**Keywords:** laser cladding deposition, 12CrNi2 alloy steel powder, substrate preheating, microstructure and properties, residual stress

## Abstract

The 12CrNi2 alloy steel powder studied in the present paper is mainly used to manufacture camshafts for nuclear power emergency diesel engines. Laser cladding deposition is of great significance for the manufacture of nuclear power emergency diesel camshafts, which has the advantages of reducing material cost and shortening the manufacturing cycle. However, due to the extremely uneven heating of the components during the deposition process, a complex residual stress field occurs, resulting in crack defects and residual deformation of the components. In the present paper, 12CrNi2 bulk specimens were prepared on the Q460E high-strength structural steel substrate at different preheating temperatures by laser cladding deposition technique, and a finite element residual stress analysis model was established to investigate the effects of different preheating temperatures on the microstructure, properties, and residual stress of the specimens. The results of the experiments and finite element simulations show that with the increase of preheating temperature, the content of martensite/bainite in the deposited layer decreases, and the ferrite content increases. The proper preheating temperature (150 °C) has good mechanical properties. The residual stress on the surface of each specimen decreases with the increase of the preheating temperature. The longitudinal stress is greater at the rear-end deposition part, and the lateral residual stress is greater on both sides along the scanning direction.

## 1. Introduction

Traditional technologies for the manufacturing of nuclear power emergency diesel engine camshafts are forging machining, post-forging heat treatment, etc. During machining of such parts, due to very dense surfaces, loose cores, insufficiently matched toughness, and forging cracks on the surface, manufacturing of nuclear power emergency diesel generator camshafts need to take new preparation methods into account. The 12CrNi2 metallic powder-based laser cladding deposition studied in the present paper will be mainly used for the production of camshafts for nuclear power emergency diesel engines, which provides a theoretical basis for ensuring safe operation and independent development. From a report by Murr et al. [1], it is known the recent progress has been made in the characterization and analysis of AM (additive manufacturing) prototypes fabricated by laser and electron beam melting technologies, referred to as direct metal laser sintering (DMLS), or selective laser melting (SLM) and electron beam melting (EBM), respectively [1]. Depending on the characteristics of high-energy density and non-contact processing in laser cladding deposition techniques, it can be used to effectively increase the material quality of refractory alloys, titanium alloys, nickel-based superalloys, and refractory metal materials; this has brought tremendous contributions to the aerospace, rail transit equipment manufacturing, and biomedical manufacturing industries [2]. The Hanover Laser Research Center in Germany selected the induction preheating method to study the direct forming of superalloy laser metals, which can effectively eliminate the defects of deposit cracking, and can be successfully applied to the repair and forming of superalloy blades. The Los Alamos Laboratory, Sandia Laboratory, and EADS have respectively optimized the process of laser metal deposition specimens such as ferroalloys and titanium alloys and produced fully dense and defect-free products with higher properties than forgings [3,4]. Ge et al. [5] have studied the effects of different laser power, scanning speed, and powder feeding rates on the forming quality and dimensional accuracy of DZ125L superalloy. From three aspects including the ratio of depth to width, laser energy density, and material dilution ratio, a characterization method of three-dimensional cladding layer was proposed, which provides a theoretical basis for the shape control of laser metal direct forming [5]. Laser cladding deposition technique is advantageous because of its high-machining precision, high-economic gain, simple modeling, short machining cycle, etc., so it holds great promise for research. Laser machining is featured by high brightness, strong directivity, and high-energy density. It can instantly form a high-temperature laser molten pool. However, due to the local heating of the substrate surface and the constant movement of the heat source, the overall heating of the component is extremely uneven. Solid–liquid phase changes are repeated in the laser molten pool. The thermal expansion during the formation of the molten pool produces a scompressive stress subject to the constraints of the surrounding cold end zone. During the cooling of the material after solidification, the metallic iron volumetrically changes through allotropic transformation to form structural stress. The superposition of the two force fields leads to a complicated residual stress field, which causes defects such as cracks and residual deformation [6]. For the residual stress problem of laser metal direct forming, substrate preheating is an effective stress control method for increasing the laser absorption rate of metal materials, reducing temperature gradients and cooling rate, and improving defects such as cracks. Masoud Alimardani [7], University of Waterloo, Canada, and others proposed a three-dimensional transient finite element analysis method. The stress field and temperature field of a 304 stainless-steel thin-walled wall after preheating and specimen restraint were studied. The multi-layer material addition of a 304 stainless-steel thin-walled wall was simulated, and the transient temperature distribution of molten pool and the real-time evolution of a stress field were obtained.

In the present paper, the substrate was preheated at different temperatures. The changes in microstructure, properties, and residual stress of the materials under different preheating conditions were explored, which effectively improved the defects due to the overall unevenness in heating during laser cladding deposition, and enhanced its performance to a certain extent while being applied in the process of manufacturing of nuclear power emergency diesel engine camshafts by laser cladding deposition.

## 2. Experimental Material and Methods

The experimental material was 12CrNi2 low-alloy steel powder with a particle size of 106 to 180 μm. The substrate material was a Q460E low-alloy, high-strength steel plate with a thickness of 10 mm, according to standard GB/T 1591-2018; the mechanical properties are shown in Table 1. The national standards for the chemical composition and mechanical properties of 12CrNi2 powder are shown in Table 2 [8].

**Figure 1:**
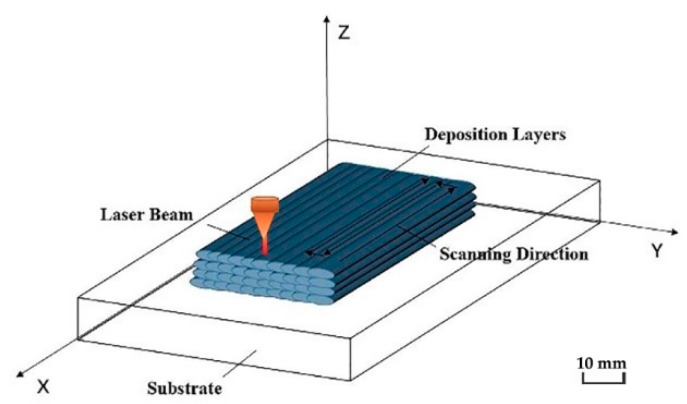
Schematic diagram of laser cladding deposition.

In the experiment, an IPG YLS-6000 laser device (IPG Photonics Corporation, Beijing, China) with a KUKA-KR30 robot control system (Keller und Knappich Augsburg, Shanghai, China) was used to control the additive manufacturing process. The main process parameters were: laser power P = 1400 W; substrate preheating temperature 20 °C, 150 °C, and 300 °C; laser scanning speed V = 8 mm/s; laser spot diameter D = 3.0 mm; powder feeding amount = 2.5 r/min (8 g/min). The powder feeding gas and the shielding gas were both argon gas (gas flow rate of 15 L/min). The scanning path was S-type reciprocating scanning along the length direction. The laser multi-pass lap ratio was 40% [9,10]. The schematic diagram of the additive manufacturing process is shown in Figure 1.

The microstructure of the deposited layer was observed using a Leica Dmi8 metallographic microscope. The microhardness of the additive specimen from the near-base layer to the surface layer was measured using a FM-700 microhardness tester (Future, Tokyo, Japan) with a load of 200 gf. A WDW-300 universal testing machine (Times Testing Instrument, Jinan, China) was used for a tensile test on the sample. The phase of the additive layer was analyzed by Empyrean X-ray powder diffraction (XRD, PANalytical, Almelo, the Netherlands). The residual stress on the surface of each test specimen was tested using a KJS-3 type indentation stress tester (Chinese Academy of Sciences, Shenyang, China).

## 3. Test Results and Analysis

### 3.1. Microscopic Analysis

**Figure 2:**
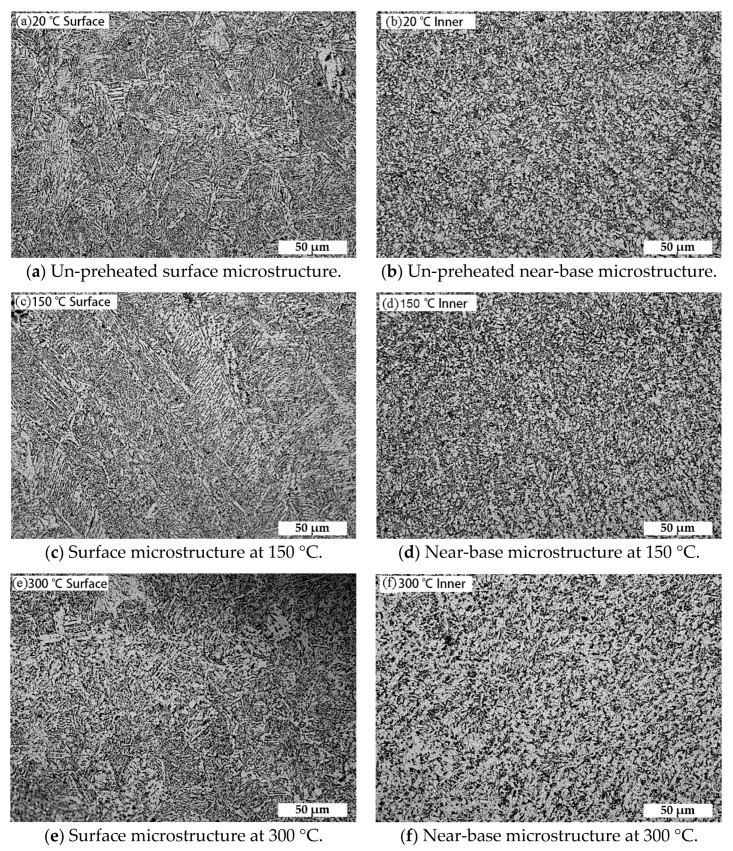
Microstructure at different preheating temperatures.

Figure 2 shows the microstructure of the surface layer and the near-base layer of the deposited layer at different preheating temperatures. The surface microstructure of each test specimen consists of martensite, upper bainite, and acicular ferrite. As the preheating temperature of the substrate increases, the content of martensite and bainite decreases, and that of ferrite increases. The near-base microstructure consists of granular bainite and fine-grained ferrite. With the increase of preheating temperature, the residence duration of the high-temperature zone in the thermal cycle increases, which leads to the decomposition of some granular bainite into ferrite and carbide, the decrease in the content of whole granular bainite and the increase in the content of ferrite.

**Figure 3:**
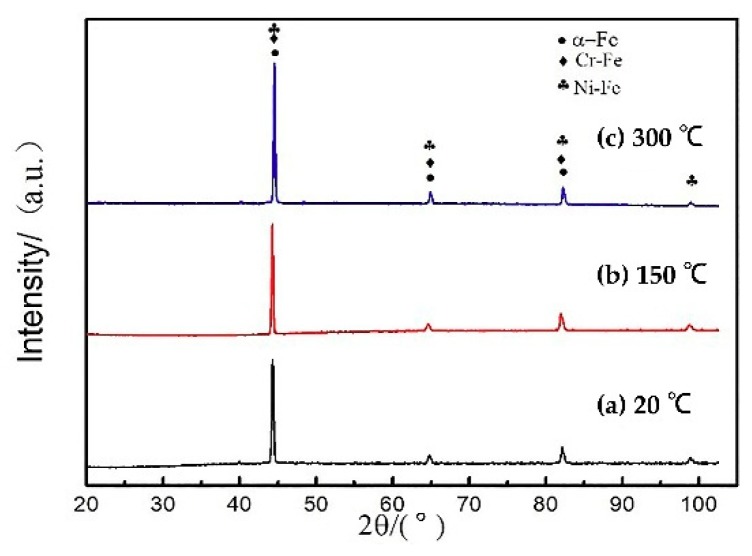
XRD image of different preheating temperatures.

Figure 3a–c shows the XRD diffraction peaks of different preheating temperature test specimens with the power of 1400 W and the scanning speed of 8 mm/s, compared with the standard PDF card, un-preheated, preheated at 150 °C, and preheated at 300 °C test specimens were consistent with the three cards of 35-1375, 34-0396, and 85-1410. It can be seen that there was no significant difference in the phase of each test specimen, which was composed of three phases of Fe, Cr-Fe, and Ni-Fe solid solution. According to the observation of the indices of crystallographic plane, the summation of the indices of crystallographic plane was even, which were all body-centered cubic lattices. With the increase of the preheating temperature, the diffraction peaks narrow and symmetrically taper, the crystallinity increases and the grain size increases.

### 3.2. Mechanical Performance Analysis

**Figure 4:**
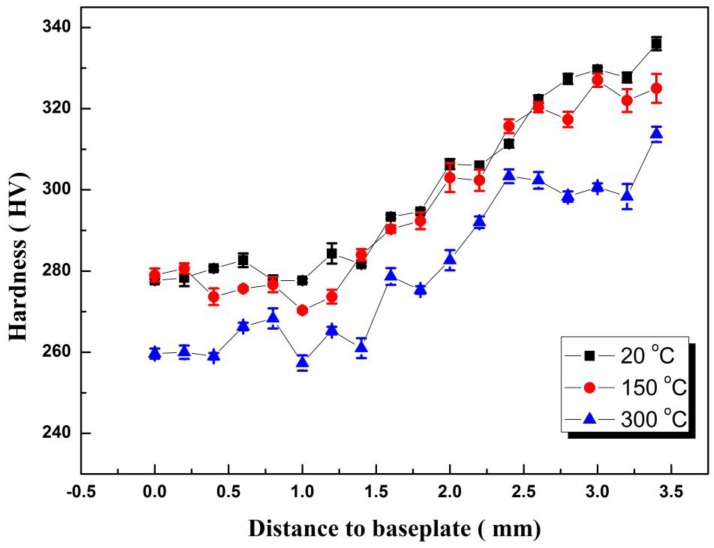
Hardness of different preheating temperature.

The hardness test position was from the surface to the inner layer, and the test interval was 0.2 mm. Then the hardness value was tested twice at the interval of 0.2 mm around each test point, and the average value of three points was taken as the hardness value of this position. Figure 4 shows the hardness distribution of the cross-section of each test specimen at different preheating temperatures. From the trend point of view, the hardness of each test specimen from the surface layer to the substrate was continuously decreasing. This was because the inner microstructure undergoes complex phenomena such as tempering, austenitizing, laser re-melting, and granular bainite decomposition under the repeated thermal cycling, resulting in a decrease in hardness. However, the top microstructure only undergoes a process of solidification and cooling solid-state phase transition, so the hardness was relatively high. The maximum hardness of the test specimen was 336 HV when it was not preheated. The hardness of the bottom layer of the test specimen was above 278 HV. The hardness of the test specimen preheated to 150 °C was slightly lower than that of the unheated test specimen. But when the preheating temperature rose to 300 °C, the temperature drop was slow due to the serious heat accumulation. The highest hardness of the surface layer was 313 HV, and the average hardness of the bottom layer under long-term high temperature was only 259 HV.

**Figure 5:**
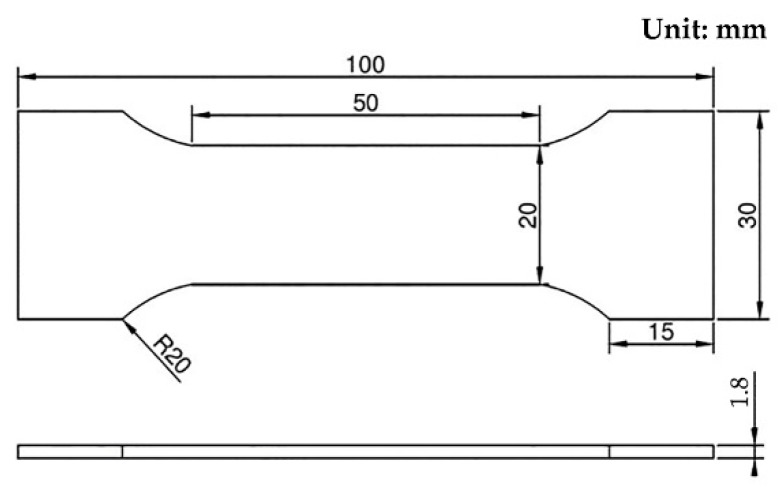
Dimension of tensile test specimen.

In the test of mechanical properties, the dimension of the additive manufacturing part of the specimen was 120 mm × 35 mm × 4 mm, and the dimension diagram of the standard tensile specimen is shown in Figure 5. Three samples were selected for each parameter test, at last, the average value was the final result.

**Figure 6:**
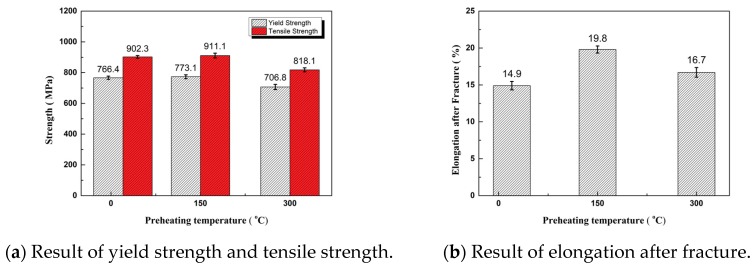
Result of the tensile properties.

By comparison among the preheated test specimens of the substrate, the test specimen preheated at 150 °C and the test specimen preheated at 300 °C, the yield strength and tensile strength of the test specimen preheated at 150 °C were the highest, and those of the test specimen preheated at 300 °C were greatly reduced—10.2% lower than those of the test specimen preheated at 150 °C. However, the yield strength and tensile strength were still higher than the mechanical properties of 12CrNi2 in the national standard. The unheated specimen had the least ductility, and the elongation after fracture was 14.9%. The test specimen preheated at 150 °C had the greatest ductility, and the elongation after fracture was 19.8%. The yield-tensile strength ratios at the three substrate temperatures were between 0.84 and 0.86, which were required by the alloy structural steel. They met the requirements of alloy structural steel and had good resistance to deformation. The test specimen preheated at 150 °C had the highest tensile strength and elongation after fracture. The results of tensile properties are shown in Figure 6.

### 3.3. Residual Stress Analysis

**Figure 7:**
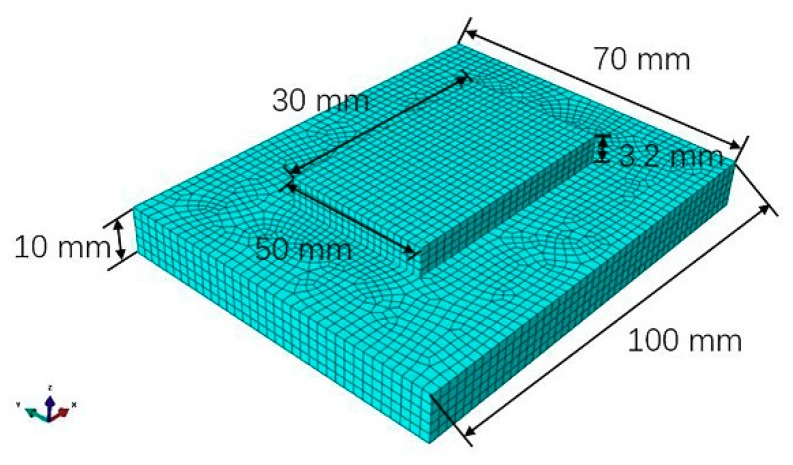
Finite element model and grid division.

The finite element analysis of temperature and residual stress field are based on ‘’Abaqus‘’ software (6.11-1, SIMULIA, Johnston, RI, USA). The model and mesh division are shown in Figure 7. The maximum mesh size of the substrate was 2 mm. The additive layer mesh size was 1 mm. The entire model had a total of 12,415 units and 15,168 nodes. The temperature field adopted the DC3D8 unit. The stress field adopted the C3D8R unit. Both units supported the dead-live unit technique, which was applicable to the finite element analysis for direct forming of laser metal powder.

In order to avoid the rigid displacement of the whole substrate and additive layer in the process of stress field calculation for direct forming of laser metal, it was necessary to impose constraints on the whole model. The finite element simulation part of the present paper applied full constraint on the bottom surface of XOY substrate. The thermophysical parameters of the 12CrNi2 material were tested by LFA (laser flash thermal conductivity apparatus) and a high temperature mechanical properties tester, (see Table 3).

**Figure 8:**
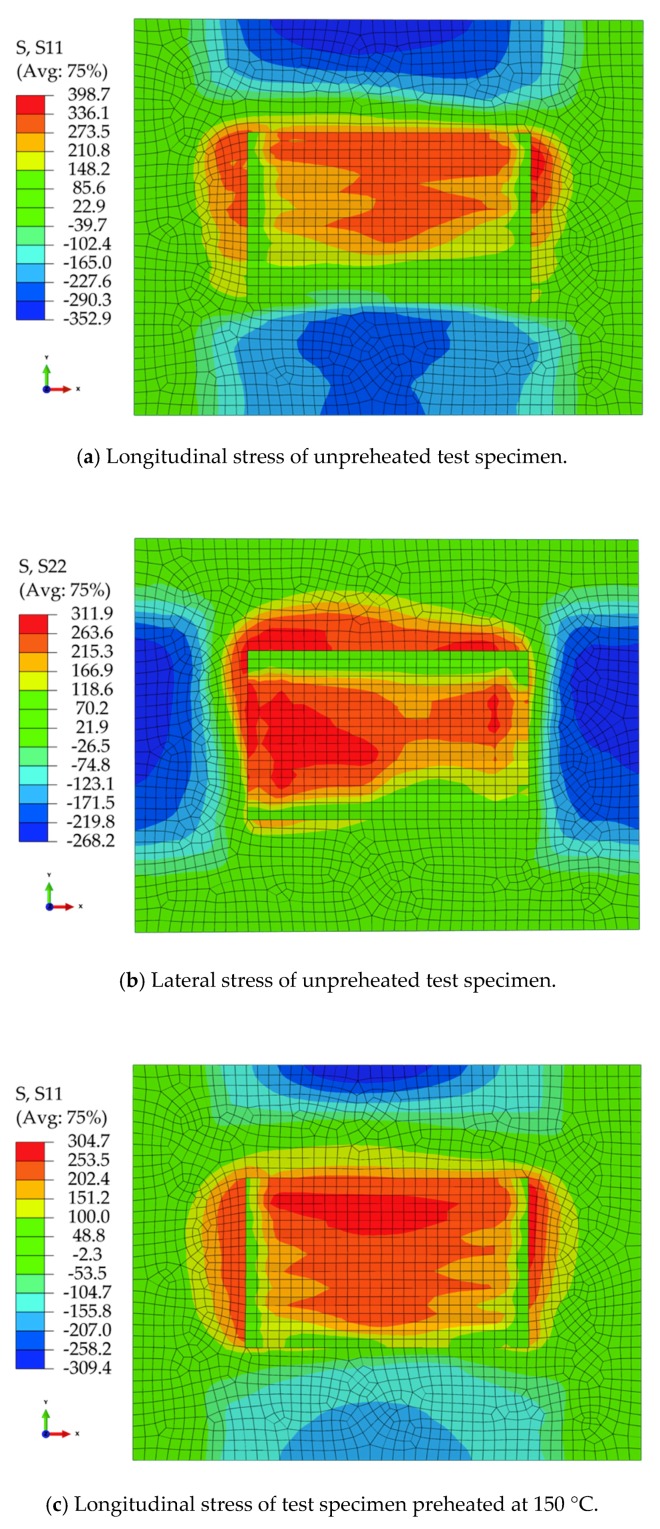

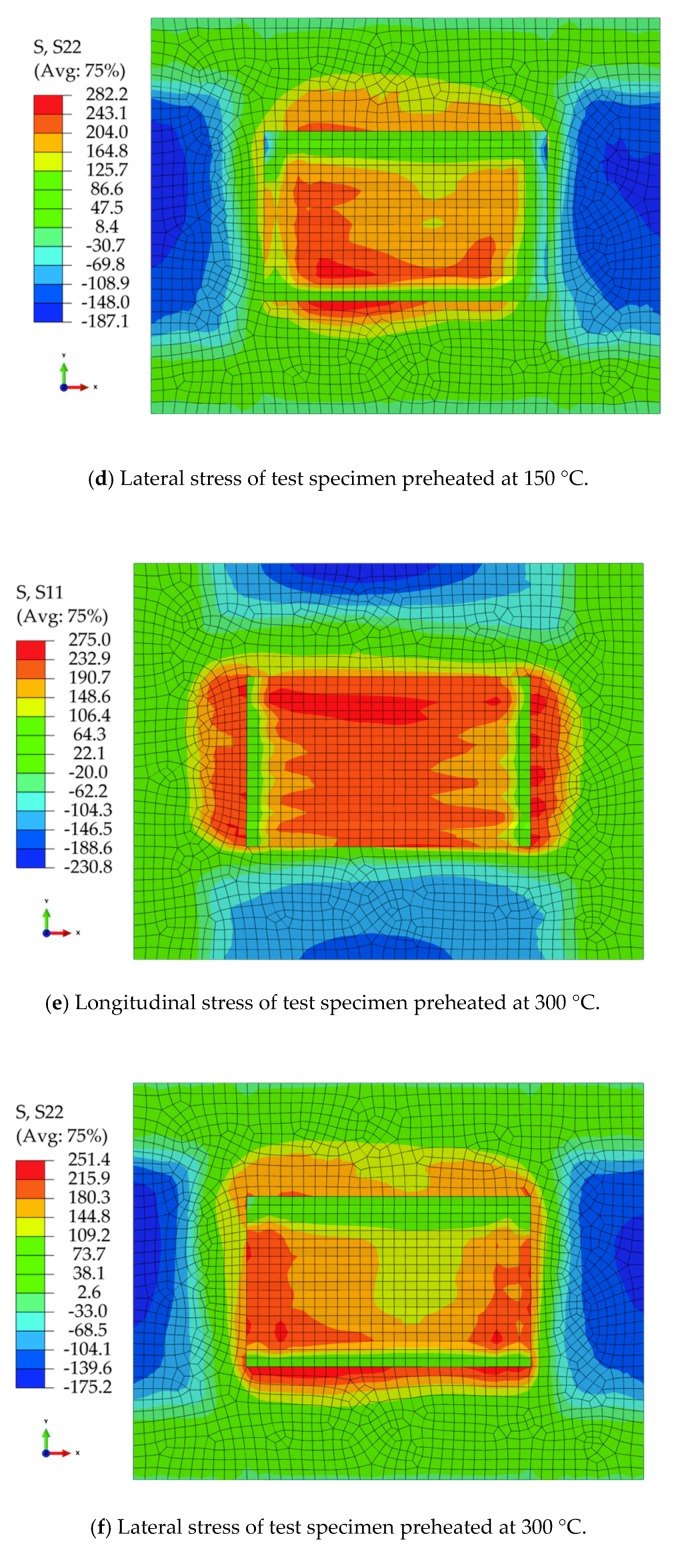
Residual stress field of different preheating temperature.

Figure 8 is a cloud diagram of the residual stress distribution on the surface of each test specimen at different preheating temperatures. It can be seen that the position of the longitudinal tensile stress on the surface of each test specimen is the end of the deposition zone. The lateral tensile stress concentration area on the surface of each test specimen is on both sides in the scanning direction. The maximum longitudinal residual stress on the surface of the unheated test specimen was 398.7 MPa, wherein the longitudinal stress in the middle of the specimen parallel to the scanning direction it was between 210.8 and 336.1 MPa. The maximum lateral residual stress was 311.9 MPa, wherein the transverse stress in the middle of the specimen parallel to the scanning direction it was between 166.9 and 263.6 MPa. The maximum longitudinal residual stress on the surface of the test specimen preheated at 150 °C was 304.7 MPa, wherein the longitudinal stress in the middle of the test specimen parallel to the scanning direction was between 151.2 and 253.5 MPa. The maximum lateral residual stress was 282.2 MPa, wherein the transverse stress in the middle of the test specimen parallel to the scanning direction was between 164.8 and 243.1 MPa. The maximum longitudinal residual stress on the surface of the test specimen preheated at 300 °C was 275 MPa, wherein the longitudinal stress in the middle of the specimen parallel to the scanning direction was between 148.6 and 232.9 MPa. The maximum lateral residual stress was 251.4 MPa, wherein the longitudinal stress in the middle of the test specimen parallel to the scanning direction was between 144.8 and 215.9 MPa. After comparative analysis, the residual stress decreased with the increase of preheating temperature.

**Figure 9:**
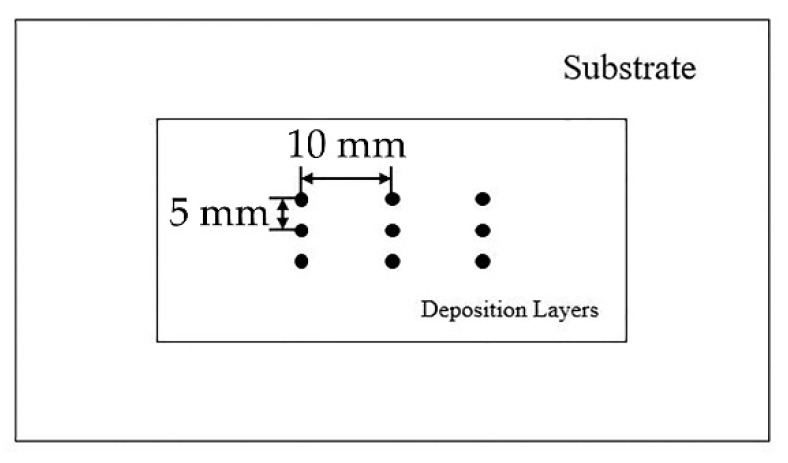
Diagram of residual stress test points.

**Figure 10:**
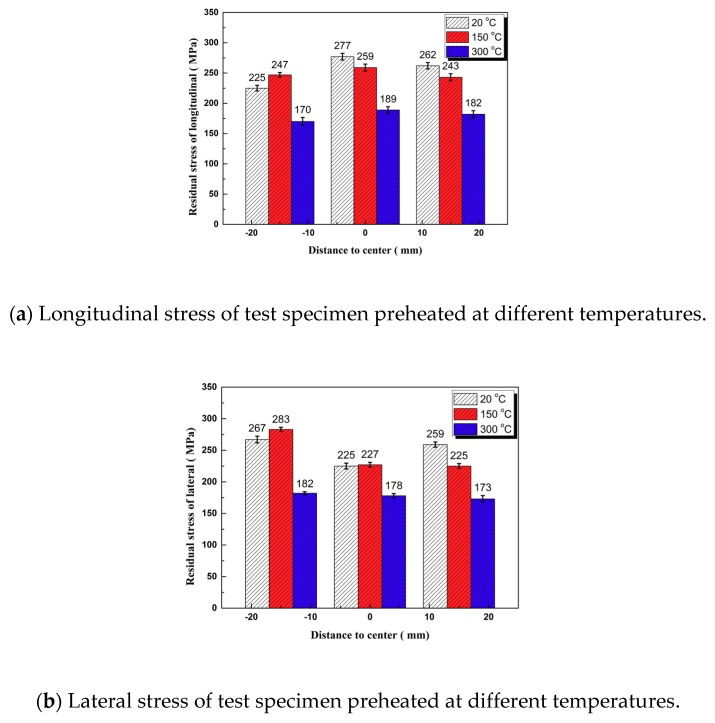
Residual stress of different preheating temperature.

The residual stress test points are shown in Figure 9. Figure 10 shows the results for testing of residual stress on the surface of each test specimens of un-preheated, preheated at 150 °C, and preheated at 300 °C. With the increase of preheating temperature, the longitudinal and lateral residual stresses were greatly decreased, and the longitudinal residual stress was slightly greater than the lateral residual stress. In full accordance with the numerical simulation law as shown in Figure 8, the numerical average deviation was 7.93%, and the simulation results agree well with the actual test values.

## 4. Conclusions

Firstly, as the preheating temperature of the substrate increased, the content of martensite and bainite in the surface layer of the deposited layer decreased, and the ferrite content increased. The granular bainite near the base layer decomposed into ferrite and carbide, and the content of bainite decreased, while the content of ferrite increased. Secondly, the hardness of the additive specimen gradually increased from the near-base layer to the surface layer, and the hardness was above 280 HV, and the maximum hardness reached 336 HV. The hardness of each test specimen decreased with the increase of the preheating temperature. When preheating temperature was up to 300 °C, due to the serious heat accumulation, the hardness decreased significantly, so too high preheating temperature could not be provided. Additionally, when the preheating temperature was 150 °C, tensile strength, yield strength, and elongation after fracture were higher than those under non-preheating. However, the properties were the lowest when preheating temperature was 300 °C. Proper preheating of the substrate can improve the mechanical properties. Lastly, the residual stress on the surface of each test specimen decreased with the increase of preheating temperature, which is based on the tensile stress. The longitudinal stress was particularly great at both ends in the scanning direction, and the lateral stress was obviously high at the back end of the deposition track.

## Figures and Tables

**Table 1:** Mechanical composition of the Q460E substrate materials.

Grade	Yield Strength (MPa)	Tensile Strength (MPa)	Elongation after Fracture (%)
Q460E	460	540–720	17

**Table 2:** Chemical composition of the 12CrNi2 materials (quality components, %).

Grade	Fe	C	Ni	Cr	Mn	Ce
12CrNi2	Bal	0.132	1.68	0.763	0.484	0.879

**Table 3:** Material parameters of 12CrNi2 steel.

Temperature T (°C)	Thermal Conductivity λ (W·m^−1^·K^−1^)	Specific Heat Capacity C (J·kg^−1^·K^−1^)	Density ρ (kg/m^−3^)	Poisson’s Ratio μ	Linear Expansion Coefficient (10^−6^/m·°C^−1^)	Elastic Modulus E (GPa)	Maximum Yield (MPa)
20	44.5	475	7850	0.28	11.3	2.05	590
300	39	550	7850	0.28	11.9	1.85	490
500	30	690	7750	0.28	12.5	1.65	410
800	22	830	7700	0.28	13.4	1.42	20
1000	22	390	7500	0.28	14.8	1.13	17
1500	21	375	7350	0.28	14.9	1.13	17
